# Human Physical Activity Recognition Using Smartphone Sensors

**DOI:** 10.3390/s19030458

**Published:** 2019-01-23

**Authors:** Robert-Andrei Voicu, Ciprian Dobre, Lidia Bajenaru, Radu-Ioan Ciobanu

**Affiliations:** 1 Faculty of Automatic Control and Computers, University Politehnica of Bucharest, 060042 Bucharest, Romania; robert_andrei.voicu@stud.acs.upb.ro; 2 National Institute for Research and Development in Informatics, 011455 Bucharest, Romania; ciprian.dobre@ici.ro (C.D.); lidia.bajenaru@ici.ro (L.B.)

**Keywords:** activity recognition, machine learning, smartphones, ambient assisted living

## Abstract

Because the number of elderly people is predicted to increase quickly in the upcoming years, “aging in place” (which refers to living at home regardless of age and other factors) is becoming an important topic in the area of ambient assisted living. Therefore, in this paper, we propose a human physical activity recognition system based on data collected from smartphone sensors. The proposed approach implies developing a classifier using three sensors available on a smartphone: accelerometer, gyroscope, and gravity sensor. We have chosen to implement our solution on mobile phones because they are ubiquitous and do not require the subjects to carry additional sensors that might impede their activities. For our proposal, we target walking, running, sitting, standing, ascending, and descending stairs. We evaluate the solution against two datasets (an internal one collected by us and an external one) with great effect. Results show good accuracy for recognizing all six activities, with especially good results obtained for walking, running, sitting, and standing. The system is fully implemented on a mobile device as an Android application.

## 1. Introduction

Recent studies (http://europa.eu/rapid/press-release_STAT-08-119_en.htm?locale=en) have shown that the number of elderly people will quickly increase in the upcoming years, which comes as a natural evolution of the fact that the median age of the general population is expected to grow (http://www.un.org/en/development/desa/population/publications/pdf/ageing/WPA2017_Highlights.pdf). From 2015, deaths in the European Union are projected to outnumber births, while almost three-times more people will be 80 or more in 2060. This will result in a growing number of older adults living alone and in need of intensive care. The social and economic point of view will be impacted by the care and assistance needs as a result of the trend of rapid growth in the number of persons with physical disabilities. On the one hand, these trends will lead to dramatic challenges for the healthcare system, state pensions schemes, and employers alike. On the other hand, they will offer innovation and business opportunities for technology providers in the field of innovative ICT-enabled assisted living or “ambient assisted living” (AAL). The main scope of such solutions is to apply the ambient intelligence (AmI) concept and technologies to help people live longer in their natural environment.

For people to remain at home, several facilities need to be offered: monitoring health status, detecting emergency situations such as debilitating falls, and notifying healthcare providers of potential changes in health status or emergency situations. The end goal is what is commonly referred to as “aging in place”, which is defined by the Center for Disease Control and Prevention as “the ability to live in one’s own home and community safely, independently, and comfortably, regardless of age, income, or ability level” (https://www.cdc.gov/healthyplaces/terminology.htm). According to a 2010 AARP survey [1], 88% of people over age 65 want to stay in their residence for as long as possible.

In this paper, we focus on one very important component of aging in place, namely activity recognition, which represents a system’s ability to recognize actions performed by users based on a set of observations of their behavior and the environment they find themselves in (being sometimes also referred to as behavior recognition). It can be employed to track the behavior of older adults and ensure that they behave in normal parameters. Furthermore, an intelligent activity recognition system can also detect when the older adults are passive and can recommend that they move around, take a walk, etc. This can be done using various senses similar to the ones humans have. Some solutions are based on computer vision [2], while other works have been based on audio recognition techniques [3] (rather a complementary addition to already existing methods) or radio frequency identification (RFIDs) [4,5,6]. Another sense suitable for human activity recognition is motion, recorded through different sensors.

Since we believe that various types of human-carried sensors might discourage older adults from participating in an activity recognition-based system, we focus on one sensor-based ubiquitous piece of technology, namely smartphones, which are far more than just communication devices. They are packed with high-end hardware and features for every type of user. Additionally, a large number of sensors can be found inside them, including motion sensors. Therefore, this paper studies human activity recognition and how it can be achieved using the sensors available on a smartphone.

The main objective of this paper is to recognize the type of physical activity the user is performing accurately, using the sensors of the phone. This includes analyzing the current solutions offered as a result of other research, finding ways to improve them, and a new approach for solving the stated problem. Moreover, the proposed solution is tested against a newly-collected dataset, as well as an already existing one. As for further development, secondary objectives aim to make the phone react in an appropriate way for each kind of activity, meaning that when one of the activities is detected, the phone will take an action or will notify the user with helpful pieces of advice in the given situation, depending on the settings.

The proposed approach implies developing a classifier using three sensors available on a smartphone (accelerometer, gyroscope, and gravity sensor), while adhering to the best machine learning (ML) practices. This involves collecting a relatively large dataset for training the classifier, extracting features from the data, and using an ML algorithm to classify the activities, with the following options being accounted for: walking, running, sitting, standing, climbing stairs, going down the stairs.

The obtained results show that human activity recognition can be successfully achieved using a smartphone’s sensors, with some activities’ accuracy reaching values as high as 94%. Some activities are more difficult to identify, as a result of the similarities between them. This is the case of climbing and going down stairs, which are often mistaken for each other or even for walking. However, the proposed solution manages to correctly identify these activities as well, although with a slightly higher error rate. Moreover, after evaluating the proposed solution against two datasets, we conclude that it can also be adapted to other recognized datasets.

The remainder of this paper is organized as follows. Section 2 explains the motivation of this paper, and then, Section 3 describes some of the related work in the area of human physical activity recognition and how it is used in real life. Section 4 further describes the proposed solution in great detail, explaining the data collection, feature extraction and the ML algorithm that was used. Section 5 describes how the solution was evaluated and reports performance results of the activity recognition algorithm, while Section 6 concludes the paper and presents future work.

## 2. Motivation

In light of recent advancements in technology, especially in domains like artificial intelligence and machine learning, most of the gadgets people use in their day-to-day life appear not to be used to their full potential, and mobile devices are no exception.

Activity recognition is one of the many sub-domains of mobile technology that has escalated quickly over the past few years [7]. Its areas of use cover smart homes [8], fitness tracking, healthcare monitoring solutions [3,9], security and surveillance applications [10], tele-immersion applications [11], etc. Currently, with the boost in power smartphones have been given lately, most of this technology is being transferred to the mobile world. Currently, the simplest and most common usage of activity recognition on phones is represented by fitness applications, especially running tracking, a simple search on the Play Store offering tens of choices. Recently, given the whole uncertainty surrounding the security and privacy of user data, steps have been taken towards using activity recognition for user authentication.

As mentioned, multiple ways of recognizing human activity have already been suggested. Human physical activity recognition based on computer vision [2] is one of them. As an example, one solution [12] used Kinect sensors to detect skeleton data from the human body and identify the activity based on the information extracted. The authors achieved good results, correctly identifying the performed activity in approximately four fifths of the attempts. However, the main drawback of this method is the necessity of a visual sensor, which, although it has good accuracy, does not allow for too much mobility.

Other research in the area of activity recognition takes into consideration the possibility of using RFIDs to identify the action a person is executing [6]. This is done by exploiting passive RFID technology to localize objects in real time and then infer their movements and interactions. The authors were able to recognize actions such as making coffee or tea, preparing a sandwich, or getting a bowl of cereal, with probabilities higher than 90%.

Our paper targets a third type of activity recognition, which is based on motion detection using sensors found in a smartphone. This solution attempts to improve the previous two methods in terms of mobility and simplicity. As smartphones are very portable, mobility is no issue in this case, with activity recognition being available in any environment. The other target, simplicity, is also reachable thanks to the wide availability of the mobile devices. Thus, the potential of this approach is boosted by the opportunity to give people something that is very accessible and easy to use.

## 3. Related Work

During the last decade, with the new discoveries in the world in artificial intelligence and machine learning, activity recognition has received a significant share of attention. Throughout the course of these last 10 years, constant improvements have been made in this domain, early studies starting from gesture spotting with body-worn inertial sensors [13] and state-of-the-art solutions offering complex human activity recognition using smartphone and wrist-worn motion sensors [14]. In this section, we analyze some of the other methods of detecting user activity and see how they compare to our proposed solution.

### 3.1. Early Attempts

One of the early attempts to recognize human activities using sensors was through body-worn sensors such as accelerometers and gyroscopes [13], in order to detect a vast range of user activities, some of these being pressing a light button, performing a handshake, picking a phone up, putting a phone down, opening a door, drinking, using a spoon, or eating hand-held food (e.g., chocolate bars). For this, a set of sensors was placed on the arm of the tester, having one end on the wrist and one end on the upper arm, near the shoulder. The data collected consisted of relative orientation information, such as the angle the hand was being held at and the movement of the hand. The interpretation of the results was done using two metrics. The first one was recall, computed as the ratio between recognized and relevant gestures (with obtained values of 0.87), while the second one was precision, computed as the percentage of recognized gestures from all retrieved data (obtaining values of 0.62).

### 3.2. Similar Approaches

A solution somewhat similar to our proposal experimented with human activity recognition using cell phone accelerometers [15]. The authors chose to use only the accelerometer sensor because, at that time, it was the only significant motion sensor used by most mobile devices. In the mean time, several other sensors have reached the vast majority of smartphones. The activities under observation were walking, jogging, sitting, standing, climbing stairs, and descending stairs. The study was divided into three main sections: data collection, feature generation, and experimental work.

The first step, data collection, was done with the help of twenty-nine users that carried a mobile device in their trouser pocket while doing casual activities. Having also tested the possibility of 20-s stints, each activity was finally performed in 10-s stints, reading data from sensors every 50 ms. Not every user made the same number of attempts, the number of sets collected being different for each person and also for each activity performed. Because this solution implied using a classification algorithm, in the following step, the main goal was shaping the data so that they could be passed as input to existent algorithms. In this step, named feature generation, some computation was done on each set of 10-s sensor readings, resulting in six types of features: average, standard deviation, average absolute difference, average resultant acceleration, time between peaks, and binned distribution. The final step of the practical part was experimenting with the extracted features and with three classification techniques, namely decision trees, logistic regression, and multilayer neural networks. The results showed good percentages of recognizing walking (92%), jogging (98%), sitting (94%), and standing (91%), these being the average values over the three classification methods, whereas climbing upstairs and downstairs had relatively low percentages. The last two activities averaged 49%, respectively 37% over the three classifiers, increasing to 60% and 50% if the logistic regression were not taken into account. This led to the two activities being removed altogether in the end. Instead, a new activity was added for climbing stairs, with an average accuracy of 77%, still significantly lower than what was obtained for the other four activities, but more reliable.

In comparison to the solution proposed in this paper, this approach had the following drawbacks: it used less sensors (only one, compared to our four); the data were collected while holding the device in just one position (compared to five in our case); and the accuracy for activities involving stairs was lower than what we managed to obtain, as shown in Section 5.

A survey of similar approaches was performed in 2015 [16], where the authors analyzed activity recognition with smartphone sensors. They categorized the type of sensors existing in smartphones (accelerometer, ambient temperature sensor, gravity sensor, gyroscope, light sensor, linear acceleration, magnetometer, barometer, proximity sensor, humidity sensor, etc.), as well as the types of activities that can be recognized, ranging from simple activities like walking, sitting, or standing, to more complex ones such as shopping, taking a bus, or driving a car. Furthermore, activities can also be split into living activities (eating, cooking, brushing teeth, etc.), working activities (cleaning, meeting, taking a break, working, etc.), or health activities (exercising, falling, following routines, etc.). Very importantly, the authors also extracted some challenges related to smartphone-based activity recognition, among which are subject sensitivity, location sensitivity, activity complexity, energy and resource constraints, as well as insufficient training sets.

### 3.3. Multi-Sensor Approaches

One of the more recent and complex studies proposed a solution based on both smartphones and wrist-worn motion sensors [14]. The main idea of this approach is that the way smartphones are held by their users (e.g., in the trouser pocket) is not suitable for recognizing human activities that involve hand gestures. That is why additional sensors are used besides the ones from the device. Both sets of sensors included the accelerometer, gyroscope, and linear acceleration.

The data used were collected for thirteen activities, from ten participants, but only seven of those activities were performed by all the participants, which are exactly the activities that the proposed solution is aiming to recognize. Each activity was performed for 3 min, the total amount being 30 min for each activity, resulting in a dataset of 390 min. All data collection was performed by carrying two mobile phones. Therefore, instead of using wrist-worn sensors, a second smartphone was placed on the right wrist of the users. Only two features were extracted, namely mean and standard deviation. These have been chosen for the low complexity and the reasonable accuracy shown for various activities. The results were composed of combinations of sensors. Each sensor was evaluated alone and then in combination with the other ones and also combining the two positions for the mobile devices. There were a few errors in recognizing the activities when using only the accelerometer and gyroscope at the wrist position, the biggest confusion being between walking and walking upstairs, with an accuracy of 53%, thus averaging 83% together with the other activities. The results were improved when combining the two test positions, the overall accuracy increasing to 98%. The main drawback of this solution, when compared to the proposed solution, was the use of two mobile devices, which is unfeasible in real life.

An interesting project that also used multiple sensors from Android devices is Social Ambient Assisted Living or SociAAL (http://grasia.fdi.ucm.es/sociaal/), where virtual living labs were created using a framework specifically designed for this purpose, called PHAT (Physical Human Activity Tester) [17]. The devices in the project used Android and were able to take advantage of all sensors that can be found on such devices, including camera, microphone, accelerometer, user input, etc. [18].

### 3.4. Fitness

One area that has resonated greatly with activity recognition lately is sports, especially fitness and running. There are countless examples of applications that use human activity recognition to help users track their training sessions. Samsung Health (https://play.google.com/store/apps/details?id=com.sec.android.app.shealth) offers such a tool beside many more, but there are other apps that focus entirely on running and walking, like Nike+ (https://play.google.com/store/apps/details?id=com.nike.plusgps) and Endomondo (https://play.google.com/store/apps/details?id=com.endomondo.android), or even cycling and swimming like Strava (https://play.google.com/store/apps/details?id=com.strava). Although these apps recognize a very limited number of activities, they have excellent results, being extremely accurate in detecting the type of activity performed, offering their clients benefits like auto pause when they detect the user is not running anymore and personalized training patterns.

Furthermore, in recent years, there has been a significant growth in the amount of smart watches and fitness bands such as Fitbit (https://www.fitbit.com/) and Apple Watch (https://www.apple.com/lae/apple-watch-series-4/), which are able to track the number of steps, sleep patterns, passive periods, etc. These kinds of devices have started being used as components of more complex systems that employ many sensors to perform in-depth activity recognition for scenarios such as healthcare [19], healthy aging [20], persuasive technology for healthy behavior [21], etc.

### 3.5. Human–Computer Interaction

People’s pleasure in tendency and to play never disappears, and there have been many improvements in the gaming area. In the past few years, activity recognition has become an active part of playing, with the creation of technologies like Kinect [12], PlayStation Move [22], and Nintendo Wii [23]. While some of these recognize activity only by using visual computing (like Kinect), the others also rely on sensors. The Nintendo Wii has a remote that has a motion sensor that makes the activity recognition possible. All these are used in different ways to play games and even to create a healthy habit. By recognizing the activities a user is performing, the computer is able to understand the user and give a response based on human reactions. This way, the human–computer interaction is possible.

### 3.6. Healthcare Monitoring

Recent studies [24] have shown that user activity and behavior can be used to indicate the health status of humans. Investigations have led medical experts to the conclusion that there is a strong correlation between the amount of physical activity and the different diseases related to obesity and metabolism. Considering the great quantity of data that can be collected regarding a person’s activity, the idea of using these data to gather information about the human medical condition has grown rapidly. Although it is believed that this may be a better solution than a time-limited medical appointment, it is not regarded as a replacement, but more like an additional tool.

One of the earliest attempts to tackle this problem [25] was made using a Bluetooth-based accelerometer worn in three positions and RFID modules used to tag objects used daily. The paper concluded that most people stay in one of the following states: sitting, standing, lying, walking, and running. Furthermore, the described solution included a wrist-worn sensor used to detect hand activity in order to improve the accuracy of human activity recognition. This sensor is also fitted with an RFID reader to detect tags placed on certain objects. When the sensor reads a nearby tag, it offers data describing the hand motion when using that object.

For the evaluation part, an analysis was made over several activities coupled with each body state like drinking while sitting, standing, or walking and ironing, or brushing hair while standing. The results showed a significant accuracy improvement with the use of RFID, the overall value increasing from 82%–97%. When taking into account only the body state recognition, the results averaged 94%, labeling walking as the hardest activity to identify.

## 4. Proposed Solution

This section contains a step-by-step description of the proposed solution. Section 4.1 presents the process of collecting the dataset and the testing data. Section 4.2 explains the features extracted from the data, while Section 4.3 offers an overview of the classification algorithm.

### 4.1. Data Collection

The data collection can be separated into two groups: training data and testing data. Moreover, the testing data collection is also divided in half: one part for the data we gathered ourselves using an application specifically created for this purpose (the “internal dataset”) and the other one for an external dataset [26]. This partitioning of the testing data was necessary because the two datasets used data from different sensors (as shown in Table 1).

The data collection Android application offers multiple customization options, while the default values set are the ones the the proposed solution uses for the data in the training dataset. Regarding activity selection, the user can choose between walking (default value), running, sitting, standing, and going up or down the stairs. Moreover, the user can also set the sensors’ reading interval (which is set to 50 ms by default) and the sample recording duration (set to 10 s). Finally, users are also able to choose the sensors that will collect data, from accelerometer, gyroscope, linear acceleration sensor, magnetometer, and gravity sensor (by default, only the first four are enabled).

To save time and resources, this application is used for feature generation as well. The process of extracting features from the collected data is described in Section 4.2. For testing purposes, this application also has implemented capabilities of activity recognition. After collecting a set of data from sensors, activity recognition based on that data can be triggered from a button, the result being returned to the user with a Toast.

**Figure 1:**
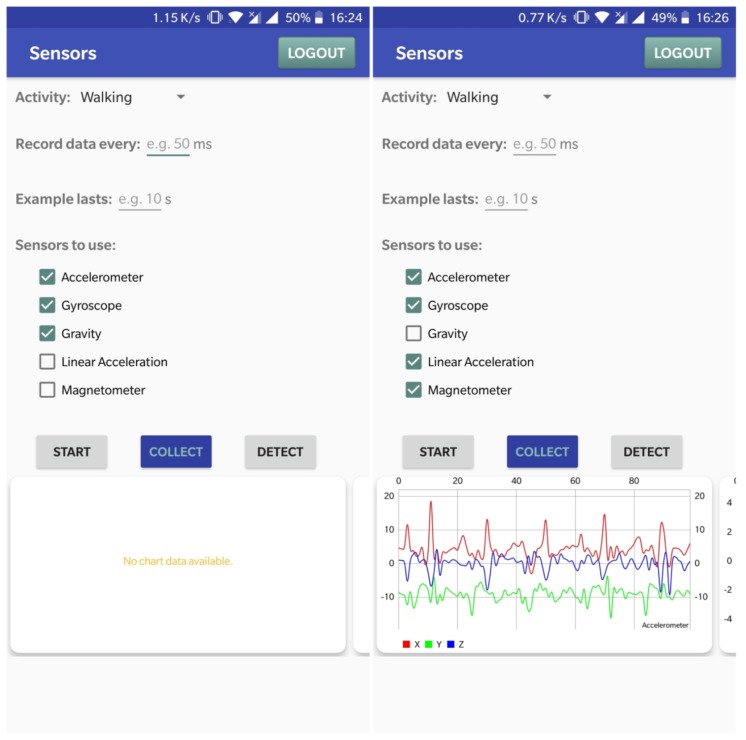
Main screen of the data collection application, before and after recording data.

For the internal dataset collection, five participants were involved, whose role was to help collect usage data by carrying their smartphones while performing the following activities: walking, running, sitting, standing, climbing stairs, and going down the stairs. During these activities, the Android data collection application (shown in Figure 1) collected data from three sensors: accelerometer, gyroscope, and gravity sensor. Because of the way machine learning algorithms work, reading a large amount of data at once was not an option, so, as suggested in [15], each entry in the dataset was created using 10-s segments. For each segment, our app recorded raw data from the sensors, each sensor providing three axes: *X*, *Y*, *Z*. The main difficulty of this part was caused by the fact that the Android platform does not allow reading data from a sensor at an exact moment in time. In fact, the data can be read only when a change is detected in the sensor reading. On top of that, different sensors report data at different times, and only one sensor reading can be accessed at a time. To overcome these drawbacks, the proposed solution calculated the average number of readings for each sensor (as shown in Table 2) and, by taking a margin of error, kept only the first 100 readings from each sensor per 10-s interval. In the end, each 10-s segment provided a set of 900 readings (300 for each sensor, 100 for each axis).

The collected data were written to a file stored locally on the phone’s internal storage. When the sensors listener detects a change, it checks which sensor triggered it and saves the readings in the corresponding list. This step is repeated during the whole recording period for each change in the sensor’s values. When the time is out, only one hundred values per axis are kept, with three axes per sensor. Then, features were extracted from the data, and they were written to the file as one line, one after another.

### 4.2. Feature Extraction

After collecting the data, it had to go through a transformation process in order to extract features that provide all the necessary information to the algorithm used for ML. For every set of readings, we computed five types of features, each generating a number of inputs for the learning algorithm. A brief description of the features can be found below.

#### 4.2.1. Average

There were nine inputs for this feature, which represented the average value of readings per axis, computed as follows (where *N* is the number of readings for each sensor per 10 s, for this and all the following equations):

(1) $$\frac{1}{N}\sum_{i=1}^{N}x_{i}$$

#### 4.2.2. Average Absolute Difference

This feature (also with nine inputs) is the average absolute difference between the value of each of the readings and the mean value, for each axis, computed as:

(2) $$\frac{1}{N}\sum_{i=1}^{N}\left|x_{i}-\mu \right|$$

#### 4.2.3. Standard Deviation

The standard deviation was employed to quantify the variation of readings from the mean value, for each axis (resulting in nine inputs):

(3) $$\sqrt{\frac{1}{N}\sum_{i=1}^{N}x_{i}-\mu }$$

#### 4.2.4. Average Resultant Acceleration

This feature, having the inputs, was computed as the average of the square roots of the sum of the squared value of each reading:

(4) $$\frac{1}{N}\sum_{i=1}^{N}\sqrt{{x_{i}}^{2}+{y_{i}}^{2}+{z_{i}}^{2}}$$

#### 4.2.5. Histogram

Finally, the histogram implies finding the marginal values for each axis (minimum -maximum), dividing that range into ten equal-sized intervals and determining what percentage of readings fall within each of the intervals (resulting in 90 inputs):

(5) $$\frac{1}{N}\sum_{i=1}^{N}\left[\left(x_{i}\;in\;b_{j}\right)\to 1,j=1\ldots 10\right]$$

### 4.3. Classification Algorithm

Many machine learning solutions have been employed in activity recognition [9], including C4.5 decision trees, RIPPERdecision rules, naive Bayes classifiers [27], support-vector machines (SVMs) [28], random forests, bootstrap aggregating (bagging) [27], adaptive boosting (AdaBoost), k-nearest neighbor classifiers [27], hidden Markov models [29], etc. For the training of our classifier, the proposed solution used a multi-layer perceptron (MLP) [30]. The choice of using this type of neural network was made in accordance with several factors:

- simplicity—the MLP is a feedforward neural network, which means there is no cycle between the nodes, making it one of the simplest types of neural networks, so it is very easy to create and use;
- resource consumption: due to its simplicity, this classifier is very cost-effective; given the fact that needed to run on a mobile device and the resources were limited, the results to resource consumption ratio is very high;
- input: the restrictions related to the input are very permissive, the perceptron accepting a wide range of input types;
- output: the network outputs the probabilities of every possible class.

The MLP used in this project consisted of three layers: an input layer, a hidden layer, and an output layer. The number of inputs was equal to the number of neurons in the input layer, and the number of outputs matched the number of possible classes, that being the activities accounted for. The network did not output an explicit class, but the probabilities associated with each activity being the one performed.

For implementing the multi-layer perceptron, we used DL4J (https://deeplearning4j.org), a Java library used to configure deep neural networks made of multiple layers, with only a small effort of configuration. The main feature that DL4J puts at the users’ disposal is a MultiLayerConfiguration object, which configures the network’s layers and parameters. For our MLP, the input layer was the starting layer, having 120 inputs representing the neurons of the layer (equal to the number of features generated for each subset of data, namely 9 each for average, absolute difference, and standard deviation, 3 for acceleration, and 90 for the histogram) and 18 outputs. The hidden layer was the only middle layer of the perceptron, with 18 inputs and nine outputs. The final layer of the perceptron was the output layer, with nine inputs and six outputs, representing the final output of the network. As can be observed, except for the input layer, the number of inputs for each layer needed to be equal to the number of outputs of the previous layer. The data passed to the input layer and implicitly to the perceptron consisted of the features generated based on extracted information, as specified in Section 4.2.

Besides the layers, several other parameters have been configured for the neural network. The first parameter is the activation function, which is a node added to the output of the perceptron, used to determine the output of the network. The answer offered by the activation function can only be interpreted as a yes or a no. The type used in this implementation is the tanh function, which is a sigmoidal function whose range is in (−1,1). This was chosen because we needed to find the probabilities of each action being the one performed, and the sigmoidal function is best fit for this, having the advantages of being non-linear and differentiable. Moreover, because the lowest value is −1, the tanh activation function clearly shows which choices are incorrect, giving them negative values.

Given the fact that weights associated with the inputs are highly important, their initialization is also important, having an impact on the convergence rate of the network. The method used by our proposed network was based on the Xavier algorithm [31], which determines the “right” values based on the number of input and output neurons, thus keeping them in a fair and sensible range of values.

Another parameter strongly connected to the weights is the learning rate. It controls the adjustments of the network’s weights, the modifications being made by following the formula new_weight=existing_weight−learning_weight×gradient. Setting this parameter correctly is important because, if it is too small, the network will take too long to respond, and if it is too large, the network might return incorrect or uninformed results. The value of the learning rate in this implementation was set to 0.1.

With regards to the training of the network, a necessary parameter is the number of iterations, which represents how many repetitions are made in the training part. In each repetition, the training data were passed to the network one at a time, and the weights were modified accordingly. Just like the learning rate, this parameter has a high significance, since choosing it too small or too large will negatively affect the output. Given that the number of inputs of the neural network was relatively high, the number of iterations was limited to 100. An extra setting that is of great significance for the optimal running of the perceptron is the activation of backpropagation. This algorithm used the Delta rule [32] to find the minimum value for the cost function in order to find the best weights that helped solve the learning process.

The end result was an Android activity recognition application. It used the features generated from the data collected with the first application to train the neural network and recognize user activity based on this network. The training of the network was run only once during the lifecycle of the app. This step, as well as other initial settings were presented to the user via the UI as a loading screen. Once all the preparations were done, users could make a few personalized settings such as how often they wanted the activity to be recognized or if notifications should be sent to inform about the result. If the latter was checked, every time an activity was recognized, a notification would be sent on behalf of the application, asking the user if the prediction was good or not. The user’s answer to the notification was used internally to improve the accuracy and also keep a record of the results.

## 5. Results

This section presents the results obtained by the current solution and offers a thorough analysis and evaluation. Section 5.1 contains charts with data read from each sensor, for every type of action. Then, in Section 5.2, we compare the results obtained when testing with the two available datasets. Finally, Section 5.3 consists of a brief analysis of the results, while also examining the possibility of improving them. It should be mentioned that the system on which the solution was implemented and tested had an Intel Core i7-7700HQ CPU clocked at 2.80 GHz and 8 GB of RAM. Thus, it had enough processing power to facilitate the use of more resource-consuming technologies, but the limited amount of memory restricted the size and depth of the neural network. Furthermore, the Android application was run on a high-end OnePlus 5T device, which was one of the best performing smartphones at the time of writing this paper, having no problem in running even the most heavy of applications.

### 5.1. Data Interpretation

As mentioned in Section 4.1, the two datasets used did not contain data from exactly the same sensors, but because the graphical shape of the data was the same in both of them, this section presents the testing data, which were the same regardless of the training data. The following charts were also offered in the application when users requested collecting new data. In the following sections, besides the interpretation of each chart independently, a comparison of the similarities and differences is offered where necessary. For each action, the figures present (in this order) data from the accelerometer, gravity sensor, gyroscope, linear acceleration sensor, and magnetometer. Although both datasets contained data recorded while holding the device in different positions, the charts presented here were generated while holding the device in the right trouser pocket, oriented downward and facing the inside of the trouser.

#### 5.1.1. Walking

**Figure 2:**
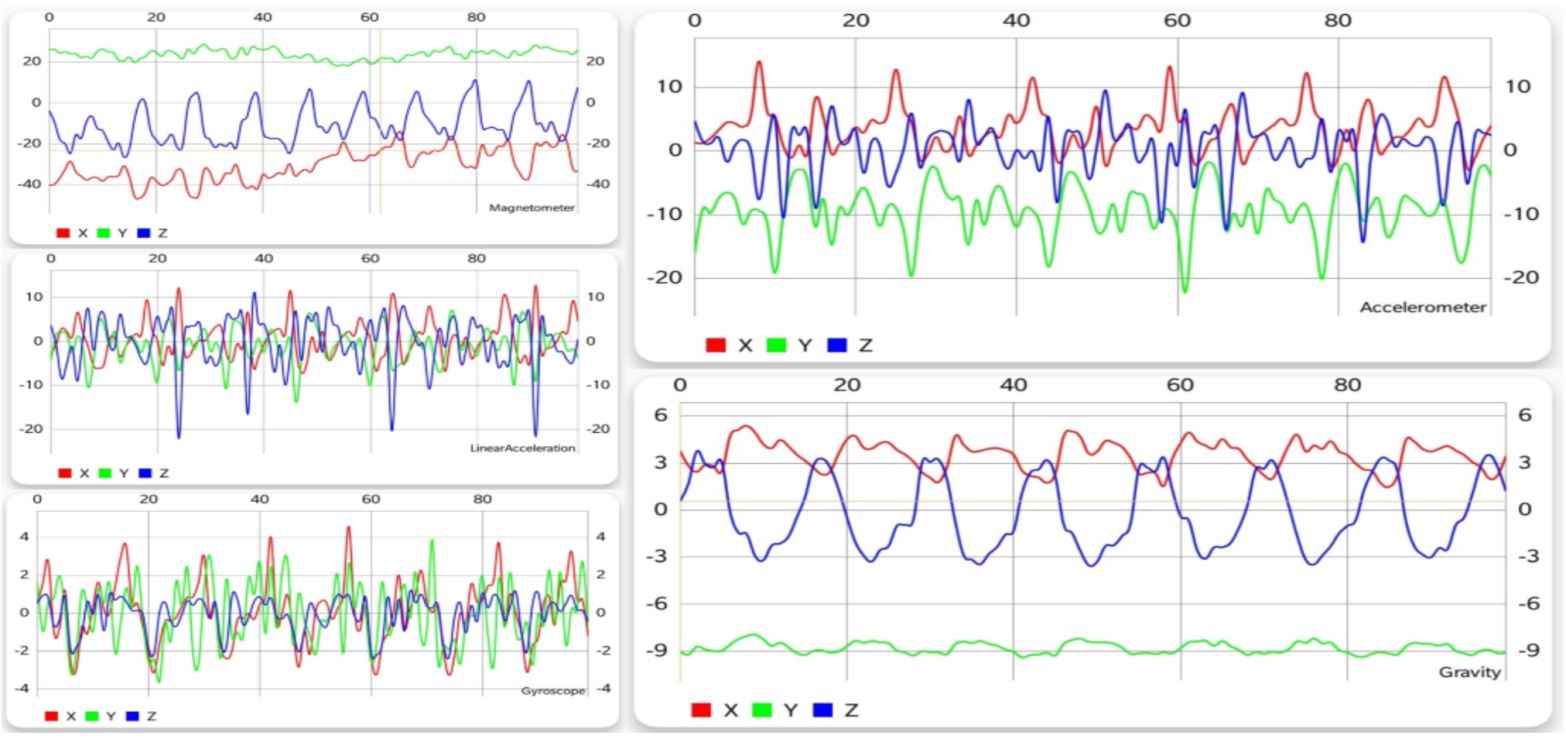
Graphical data gathered from each sensor while walking.

As can be observed in Figure 2, walking generates a periodic pattern for each of the sensors, thus offering a large amount of information to the learning algorithm, so that the classifier can distinguish it from other activities easier. Of course, this is just one possible pattern, and depending on characteristics like age, gender, height, or weight, it can vary slightly. However, this affects only less significant metrics such as the interval of the repetitions or the range of values, but the shape and pattern of the movement is almost the same.

#### 5.1.2. Running

**Figure 3:**
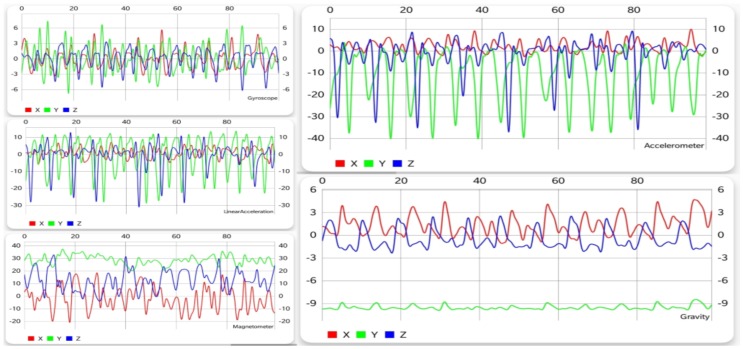
Graphical data gathered from each sensor while running.

Running implies a motion similar to walking, but executed faster. Looking at Figure 3, a periodic pattern can also be noticed for this activity, with a shorter time difference between periods for running than for walking. Although the patterns for the two activities have some common attributes, a clear difference can be seen from each sensor, especially the accelerometer and gravity sensor.

#### 5.1.3. Sitting

**Figure 4:**
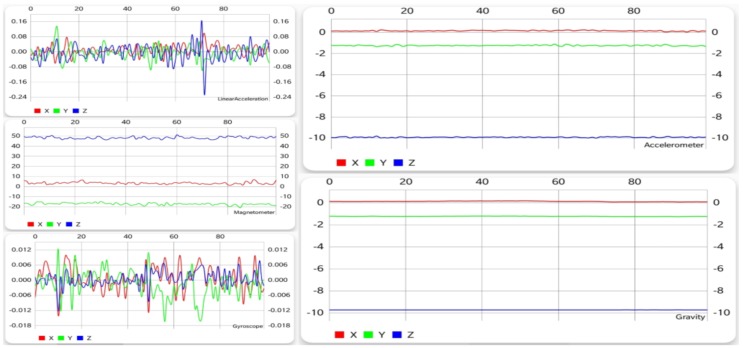
Graphical data gathered from each sensor while sitting.

Unlike the previous two activities, sitting is a motionless activity, if it can be called an activity at all. Figure 4 shows that, with the exception of the gyroscope and the linear acceleration sensor, all other sensors display little change in the values of each of the three axes, the accelerometer and gravitational data being almost unchanged. Even the first two mentioned sensors show much less fluctuation than walking and running, thus making sitting very easy to differentiate from them.

#### 5.1.4. Standing

**Figure 5:**
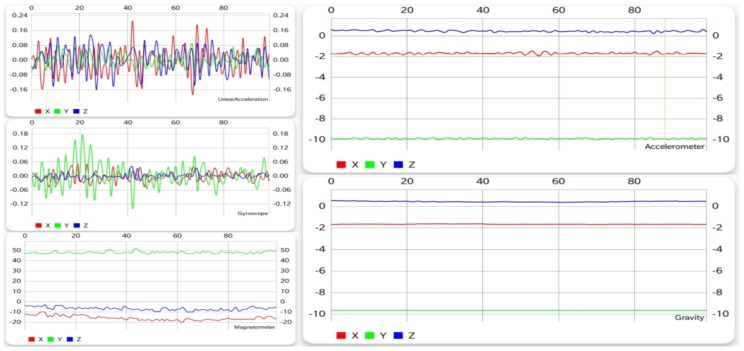
Graphical data gathered from each sensor while standing.

Standing is also an activity than involves staying still, so the charts shown in Figure 5 are similar to the ones from Figure 4. Some differences can be seen in terms of gyroscope and linear acceleration, but the main indicator when trying to distinguish the two actions is the position of the axes on the graphs. When switching from one action to the other, an interchange between the *Y* and *Z* axes can be observed. Even though these two activities are somewhat similar, they are very easy to recognize.

#### 5.1.5. Walking Upstairs

Walking upstairs is an activity that, unlike the previous ones, can be done in many ways. It is not that everyone walks or runs the same way, but with a few exceptions, the patterns they generate are more or less alike. As far as walking upstairs goes, people do it step by step, two steps at a time, some even three, slow, fast, or jumping, so recognizing it is not very straightforward.

**Figure 6:**
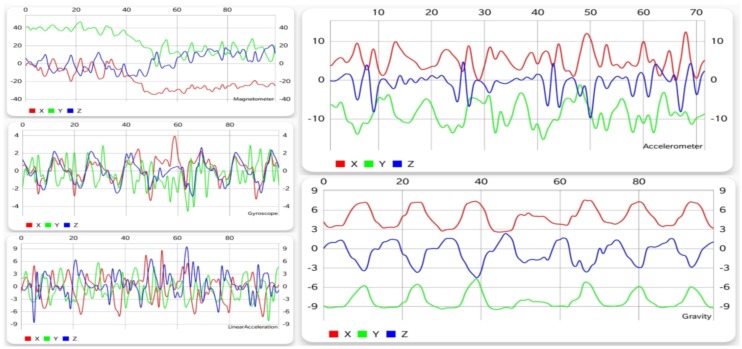
Graphical data gathered from each sensor while climbing stairs.

From Figure 2 and Figure 6, similarities can be observed between walking and climbing stairs, especially when comparing the results from the accelerometer and the gyroscope. This is partly due to the fact that there are not many stairs that take as long as 10 s to climb (since generally, one would need to spend a few seconds on the stairs, then walk on a flat surface, and so on). As described in Section 5.3, this resemblance generates confusion and some false positives, and negatives showed up when trying to distinguish and recognize these two activities.

#### 5.1.6. Walking Downstairs

**Figure 7:**
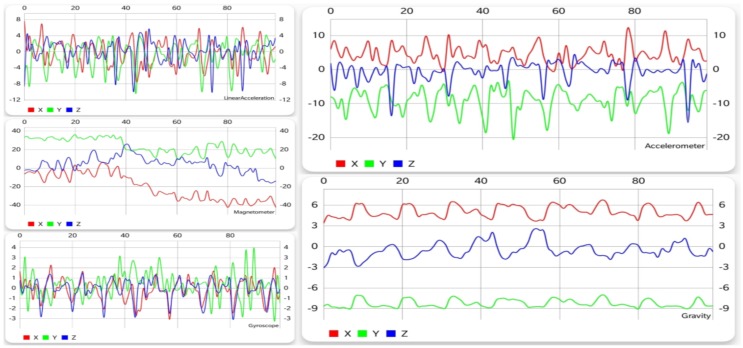
Graphical data gathered from each sensor while going down the stairs.

Just like going upstairs, walking downstairs is more complicated to detect when trying to differentiate it from other activities. Similarly to going upstairs, the patterns of going down the stairs tend to vary. Just like going upstairs, this activity is easily confused with walking. However, the biggest difficulty in recognizing both walking upstairs and downstairs is the fact that they are almost the same, which can easily be observed from Figure 6 and Figure 7, which means that the activity recognition struggled to find out which one was actually performed.

### 5.2. Datasets’ Comparison

As previously mentioned, the proposed solution was tested using two datasets, an internal one collected using our own Android application (presented in Section 4.1) and an external one [26]. The neural network was trained with each dataset, one at a time. As presented in Section 4.2, the collected dataset contained 900 examples. After extracting features from the data in the internal dataset, the total number of training examples was 15,750. For testing, the same five persons that helped with the collection of the first set were involved. The evaluation of each dataset concluded after 600 attempts, each action being performed one hundred times. The testing was done only with newly-collected data, which did not exist in any of the training sets. The confusion matrices for the two test suites are shown in Table 3 and Table 4, respectively.

By analyzing the two confusion matrices, a considerable decrease from the internal to the external dataset can be observed in the recognition of every activity, as shown in Table 5 and Table 6. With an average difference of 9.3% in favor of the internal dataset, the biggest discrepancy was found between the results obtained for running recognition, the two cases showing a difference of 13%. This can be attributed to the fact that the dataset proposed by our solution was collected in the same way and circumstances as the training examples, so the noise that affected the data was far less pronounced in this dataset than in the external one.

### 5.3. Results Analysis

On the one hand, the results obtained when evaluating the proposed solution (as shown in Table 3) with the collected dataset were satisfactory. If the activities that did not involve stairs were taken into account, the average accuracy was above 92%. Walking upstairs and going down the stairs averaged 73% in accuracy, which although not a bad result, was not considered accurate enough, especially since it was obtained on the more permissive dataset.

On the other hand, when tested against the external dataset, the correct recognition rate had a significant decrease in accuracy. All of the tested activities suffered from noise, but the effect did not vary too much over the six of them, so each activity was recognized at the same rate as the others. This means that, if sitting was the most accurately-recognized activity with the internal dataset, it remained the same with the external one. The same can be said about the other activities as well. Even with these issues, the results are encouraging, particularly for the activities that did not involve stairs. The only one of these activities to reach an accuracy below 84% was running, with 78%. Instead, the accuracy for stair ascending and descending was well below these values, averaging 63.5%. While the result is not entirely satisfactory, it should be emphasized that the external dataset used different sensors than the ones the proposed solution suggests, which may be responsible for this behavior.

A reason for the results obtained for the activities involving stairs could also be the environment in which the data collection was performed. Given that the ten-second interval for the subsets of recorded data was too long to go up or down one set of stairs only, during this process, flat zones found between the stairs may have affected the data, making the activity easier to be mistaken for walking or standing. Moreover, the lower scores of these activities are also owed to the fact that they were very much alike and were easily confused. The confusion matrices showed that most false negatives were triggered when walking upstairs and downstairs. In most cases, ascending stairs was confused with descending stairs (11.5%) and the other way around (15%), but there were also enough cases when these two activities were mistaken for walking (9%) or standing (5.2%). Concerning false positives, climbing stairs was the dominant activity, being overfitted in 29% of the cases it had been detected. Descending stairs and walking also rated high in this category, being equal at 23%. Another interesting conclusion is that the three features that had the most impact on the accuracy of the results for the internal dataset were average, absolute difference, and standard deviation, most likely because the movements that we tracked could thus be clearly differentiated per axis.

We also performed a comparison of the accuracy obtained by our solution (on both the internal and external datasets) and an external solution based on naive Bayes proposed by Shoaib et al. [14], as seen in Table 7. In this solution, the authors used motion sensors (accelerometer and gyroscope) placed on a person’s wrist or in his/her pocket and attempted to detect various activities while testing on the external dataset mentioned in Section 4.1. The comparison results are presented in Table 8, and it can be observed that our solution (Scenario 8) had the same overall accuracy as the external implementation that used an accelerometer at the user’s wrist (Scenario 1); but we were able to better detect walking (85% vs. 52%) and going up the stairs (65% vs. 48%). However, since the external dataset contained some additional activities from the ones we were focusing on (such as biking, typing, talking, smoking, etc.), there were some irrelevant data, as mentioned above. When looking at our results for our internal dataset, we can observe that they were very similar to the ones obtained by the external solution when using both an accelerometer, as well as a gyroscope (which were also sensors that we employed). The advantage of our solution comes from the fact that it was able to detect walking with a very good accuracy (93%, as opposed to 79% for the external solution), while also improving upon the “going upstairs” action. Shoaib et al. also showed in their paper [14] that using multiple sets of sensors yielded even better results (Scenario 3 in Table 8), while increasing the data collection window tended to further improve the activity recognition accuracy. This is something that we also wish to analyze for our solution in the future, and we are confident that we can obtain even better results.

## 6. Conclusions and Future Work

In this paper, we showed how human physical activity recognition can be achieved by using sensors available on a smartphone. During this work, a new dataset was collected for the six activities that made up the subject of this paper; relevant features have been extracted from the gathered data; and results were obtained using a neural network. Evaluation of the results showed that most of the activities were recognized correctly, four of them averaging an accuracy of 93%. Even with the lower scores obtained for the other two activities, the accuracy did not drop under 86%. These numbers suggest that using sensors to recognize user activity is becoming more and more reliable. The proposed solution was also tested against an external standard dataset, the results showing a slight decrease to 77%. However, the results may be affected because the external dataset contained data from some different sensors. The paper was materialized through an Android application, easily usable by any type of user, including older adults.

After achieving satisfactory results for activity recognition, we wish to take this one step further and work on a way to make the proposed solution publicly available in a form that makes it easier to be used for further research. In order to do so, a couple of actions have been planned. Because machine learning algorithms, especially deep learning ones, use many system resources, running them on a mobile device (even with the capabilities of the latest smartphones) is not a viable option. Choosing large values for the depth of the network and the size of the training dataset can easily freeze a device. Bearing this in mind, it is clear that doing all the computational part right on the device is not the best solution. Therefore, in the near future, all this heavy processing is to be shifted to a remote server. This way, the performance of the application should be improved visibly, which would make real-time detection become more of a possibility than a probability. Furthermore, we would also like to explore various alternatives to the currently-implemented perceptron classifier, such as genetic algorithms [33,34].

Another goal is to improve activity recognition by adding additional activities like riding a bike, driving, and falling. This would not only help the project cover much more of the user activity, but also offer opportunities of developing new applications that use activity recognition. One example of such an application (that we have begun working on) involves combining activity recognition with user localization, with the purpose of tracking elderly people and receiving notifications whenever their behavior is not the one expected (i.e., they go to an area of the city that they do not know; they fall; they walk in strange patterns, etc.).

## Figures and Tables

**Table 1:** Sensors registered in each dataset.

Dataset	Sensors
Internal dataset	Accelerometer, Gyroscope, Gravity
External dataset	Accelerometer, Gravity, Linear Acceleration, Magnetometer

**Table 2:** Average number of sensor readings per 10-s intervals.

Accelerometer	Gyroscope	Gravity
164	124	124

**Table 3:** Confusion matrix for the internal training set.

Performed Activity	Recognized Activity					
	Walking	Running	Sitting	Standing	Upstairs	Downstairs
**Walking**	**93**	1	0	1	3	2
**Running**	2	**91**	0	1	3	3
**Sitting**	1	0	**94**	3	1	1
**Standing**	2	1	2	**93**	1	1
**Upstairs**	8	2	1	4	**75**	10
**Downstairs**	7	2	2	6	12	**71**
**Total**	113	97	99	108	95	88

**Table 4:** Confusion matrix for the external training set.

Performed Activity	Recognized Activity					
	Walking	Running	Sitting	Standing	Upstairs	Downstairs
**Walking**	**85**	4	0	2	6	3
**Running**	5	**78**	2	3	7	5
**Sitting**	2	2	**87**	6	2	1
**Standing**	5	1	4	**84**	4	2
**Upstairs**	12	4	1	5	**65**	13
**Downstairs**	9	3	2	6	18	**62**
**Total**	118	92	96	106	102	86

**Table 5:** Activity recognition accuracy over the internal dataset.

Activity	Walking	Running	Sitting	Standing	Upstairs	Downstairs	Average
**Accuracy**	93%	91%	94%	93%	75%	71%	86.1%

**Table 6:** Activity recognition accuracy over the external dataset.

Activity	Walking	Running	Sitting	Standing	Upstairs	Downstairs	Average
**Accuracy**	85%	78%	87%	84%	65%	62%	76.8%

**Table 7:** Scenarios for activity recognition solutions’ comparison. The internal implementation refers to the solution we proposed here (using MLP), while the external implementation refers to the solution proposed by Shoaib et al. [14] that used naive Bayes. The external solution used a dataset defined in the same paper, whereas we used first our own dataset (Scenario 7) and then the external dataset.

Scenario	Description
1	External implementation, 5-s window, accelerometer at wrist
2	External implementation, 5-s window, accelerometer and gyroscope at wrist
3	External implementation, 5-s window, accelerometer and gyroscope at wrist and in pocket
4	External implementation, 2-s window, accelerometer and gyroscope at wrist
5	External implementation, 15-s window, accelerometer and gyroscope at wrist
6	External implementation, 30-s window, accelerometer and gyroscope at wrist
7	Internal implementation, 10-s window, smartphone sensors
8	Internal implementation and external dataset, 10-s window, smartphone sensors

**Table 8:** Accuracy comparison between our solution and an external solution [14].

Scenario	Walking	Running	Sitting	Standing	Upstairs	Downstairs	Overall
1	52%	100%	93%	97%	48%	74%	77%
2	79%	100%	92%	96%	74%	93%	89%
3	85%	100%	91%	92%	96%	98%	94%
4	74%	100%	94%	96%	60%	81%	84%
5	92%	100%	89%	93%	93%	98%	94%
6	100%	100%	90%	93%	97%	100%	97%
7	93%	91%	94%	93%	75%	71%	86%
8	85%	78%	87%	84%	65%	62%	77%
